# Pulmonary sequestration in adults: a retrospective review of resected and unresected cases

**DOI:** 10.1186/s12890-018-0663-z

**Published:** 2018-06-05

**Authors:** Mohammad Alsumrain, Jay H. Ryu

**Affiliations:** Division of Pulmonary and Critical Care Medicine, Gonda 18 South, Mayo Clinic, 200 First St. SW, Rochester, MN 55905 USA

**Keywords:** Pulmonary sequestration

## Abstract

**Background:**

Pulmonary sequestration (PS) is a form of congenital pulmonary malformation that is generally diagnosed in childhood or adolescence and usually resected when diagnosed. We aim to identify the clinical presentation and course of patients diagnosed to have PS during adulthood.

**Methods:**

Using a computer-assisted search of Mayo clinic medical records, we identified adult patients with PS diagnosed between 1997 and 2016. Clinical and radiological data were collected including postoperative course for those who underwent surgical resection.

**Results:**

We identified 32 adult patients with PS; median age at diagnosis was 42 years (IQR 28–53); 17 patients (53%) were men. The median sequestration size was 6.6 cm (IQR 4.4–9.3). The type of sequestration was intralobar in 81% and extralobar in 19%. The most common location was left lower lobe posteromedially (56%). Forty-seven percent of the patients presented with no relevant symptoms. The most common radiographic finding was mass/consolidation in 61% and the most common feeding artery origin was the thoracic aorta (54%). Surgical resection was performed in 18 patients (56%) and postoperative complication was reported in 5 patients (28%). There was no surgical mortality. Median duration of follow-up after diagnosis for unresected cases, most of whom were asymptomatic, was 19 months (IQR 4–26) with no complications related to the PS reported.

**Conclusions:**

Nearly one-half of adult patients with PS present with no relevant symptoms. The decision regarding surgical resection needs to weigh various factors including clinical manifestations related to PS, risk of surgical complications, comorbidities, and individual patient preferences.

## Background

Pulmonary sequestration (PS) is a congenital lung malformation that consists of a nonfunctioning lung tissue with no apparent communication with the tracheobronchial tree [1]. The blood supply to PS is through aberrant vessels from systemic circulation, most commonly the descending thoracic aorta. The term sequestration is derived from the Latin verb *sequestare,* which means ‘to separate’ and it was first introduced as a medical term by Pryce in 1964 [2, 3]. PS is rare, representing about 1 to 6% of all congenital lung anomalies and may go undetected during the prenatal period and early childhood years [4].

The PS is divided into two types, intralobar sequestration (ILS) which is the more common type, where the lesion lies within pleural layer surrounding the lobar lung and extralobar sequestration (ELS) which has its own pleural covering, maintaining complete anatomic separation from adjacent normal lung [5].

Most patients with ILS present in adolescence or early adulthood with recurrent pneumonias in the affected lobe [4]. Patients with PS can be asymptomatic and the diagnosis achieved incidentally. Other presenting symptoms may include cough, hemoptysis, chest pain and dyspnea [6, 7]. ELS rarely becomes infected because it is separated from the tracheobronchial tree by its own pleural investment [4].

There are multiple radiologic manifestations of PS on computed tomography (CT) which include mass, consolidation with or without cysts, bronchiectasis and cavitary lesions [4, 7]. Hyperlucency can be seen in ILS due to the entrance of air from the collateral drift from normal lung resulting in air trapping [4]. The arterial supply to PS is most commonly from the thoracic aorta as described for 74% of cases reported by Savic et al. in a review of 540 published cases [8]. The supplying artery may also arise from the abdominal aorta, celiac artery, splenic artery or even a coronary artery [4]. Most ILS drains to pulmonary veins while venous drainage for most ELS is to the azygos or hemiazygos vein or to the inferior vena cava [4, 8].

Most of the data pertaining to PS are from the pediatrics literature. Occasionally, PS may be diagnosed for the first time in adulthood [6, 9, 10]. Due to paucity of published data, natural history and optimal management of PS diagnosed in adults remain unclear. Furthermore, the outcome of adult patients with unresected PS is not known. Thus, we aimed to explore the clinical presentation and course of adult patients with PS including those who do not undergo surgical resection.

## Methods

Using a computer-assisted search of Mayo clinic medical records, we identified 32 adults (age 18 or greater) who were first diagnosed to have PS between 1997 and 2016. Mayo Clinic Institutional Review Board approval was obtained (#17–002077). The diagnosis was confirmed in all resected cases by histopathologic examination and the non-resected cases were diagnosed by imaging characteristics including the presence of anomalous systemic arterial supply identified by thoracic radiologists. Among the resected cases we didn’t encounter hybrid lesion of congenital pulmonary airway malformation (congenital cystic adenomatoid malformation) and PS.

Available medical records and imaging studies were reviewed to confirm the diagnosis of PS. Clinical and radiological data were collected including postoperative course in those who underwent surgical resection and the clinical course of those who did not undergo surgical resection.

### Statistical methods

Data were presented as median and interquartile range (IQR) for continuous variables and counts and percentages for categorical variables. For comparisons Mann-Whitney U test was used for continuous variables and Fischer exact test for categorical variables. Two-side *p*-value < 0.05 was considered statistically significant.

## Results

We identified 32 adult patients with PS whose median age was 42 years (IQR 28–53); 17 patients (53%) were men. The median sequestration size was 6.6 cm (IQR 4.4–9.3). The type of sequestration was intralobar in 81% and extralobar in 19%. The most common location was left lower lobe posteromedially (56%) (Table 1). The most common presenting symptom was cough (34%); however, 15 (47%) had no relevant symptoms (Table 2). Other presenting symptoms included dyspnea, thoracic pain, and hemoptysis. Recurrent respiratory infections were a presenting complaint in 16% of the patients. Asymptomatic patients had PS detected incidentally on chest imaging studies.

**Table 1:** Type and location of pulmonary sequestration (*n* = 32)

Characteristic	Number of patients (%)
Type of sequestration	
Intralobar	26 (81)
Extralobar	6 (19)
Location	
Left lower lobe	18 (56)
Posteromedial	18 (56)
Right lower lobe	14 (44)
Posteromedial	13 (41)
Anterior	1 (3)

**Table 2:** Presenting symptoms (*n* = 32)

Characteristic	Number of patients (%)
Cough	11 (34)
Chest/back pain	5 (16)
Dyspnea	5 (16)
Fever	5 (16)
Recurrent respiratory infections	5 (16)
Hemoptysis	3 (9)
Right upper abdominal pain	2 (6)
Asymptomatic	15 (47)

Note: one patient may have more than one symptom

**Fig. 1:**
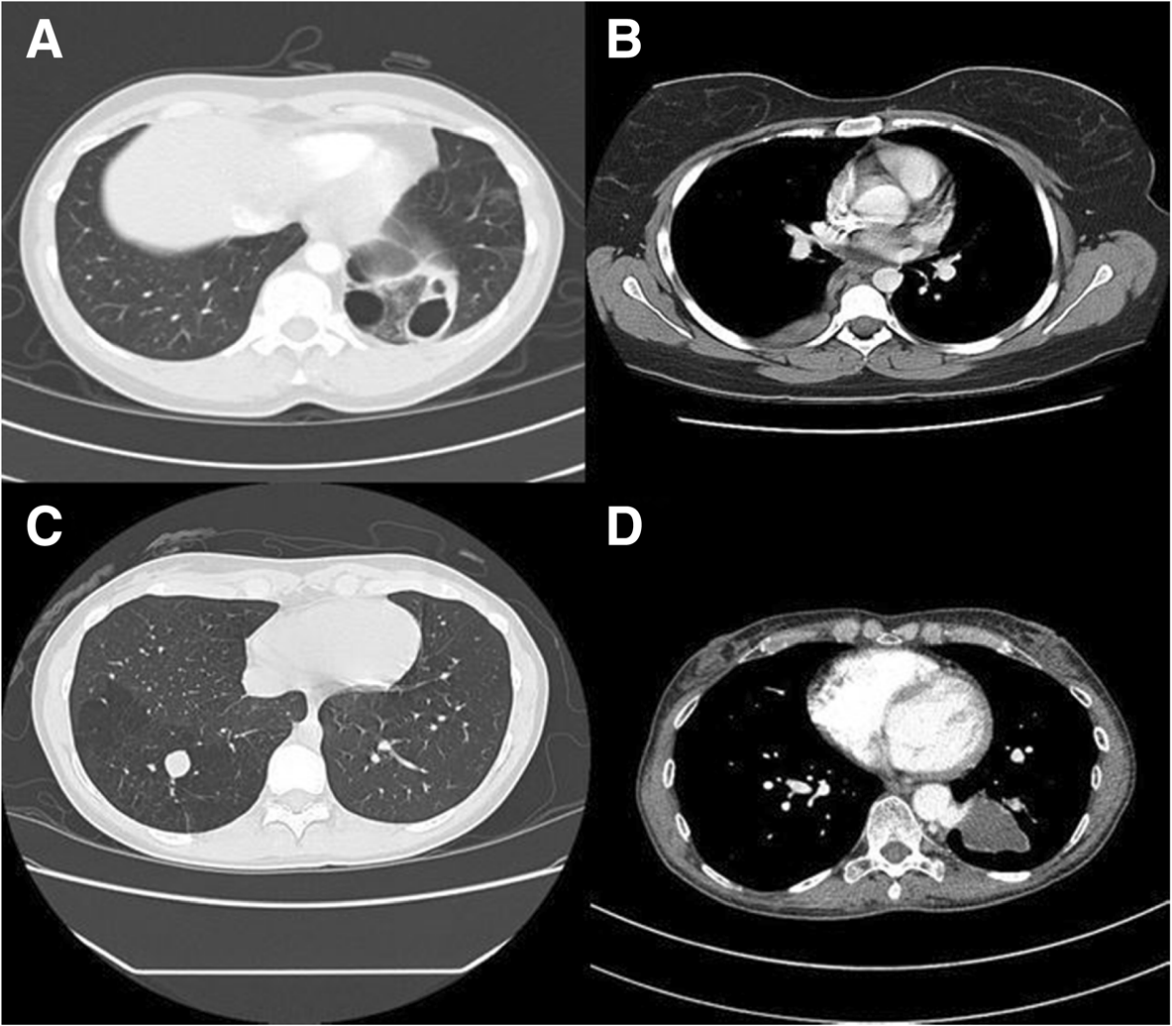
**a**: Pulmonary sequestration (intralobar) presenting as a multi-cystic lesion in the postero-basal segment of the left lower lobe. **b**: Pulmonary sequestration (extralobar) presenting as extra-pulmonary mass in the right paravertebral region. **c**: Pulmonary sequestration (intralobar) presenting as an area of hyperlucency and dilated bronchus filled with mucus in the right lower lobe. **d**: Left lower lobe sequestration (intralobar) presenting as a mass in the left lower lobe with feeding artery from descending aorta

The most common radiologic finding was mass/consolidation in 61% followed by hyperlucency in 42%; cystic changes were noted in 23% (Table 3) (Fig. 1). Dilated bronchi were seen in 15% and mixed radiologic features were in 34% of the patients. The most common feeding artery origin was the thoracic aorta (54%); others include abdominal aorta (23%), celiac (11%) and inferior phrenic/left gastric (4%). The origin of the feeding artery was not specifically identified in 2 cases (8%) both of which were resected.

**Table 3:** Radiologic manifestations (*n* = 26)

Radiologic manifestations^a^	Number of patients (%)
Mass/consolidative	16 (61)
Hyperlucency	11 (42)
Cystic changes	6 (23)
Dilated bronchi	4 (15)
Mixed features	9 (34)
Feeding artery	
Thoracic Aorta	14 (54)
Abdominal Aorta Celiac	6 (23) 3 (11)
Inferior phrenic/left gastric	1 (4)
Not determined^b^	2 (8)
Venous drainage	
Pulmonary veins	8 (30)
Azygos vein	2 (8)
Hemiazygos vein	1 (4)
Left atrium	2 (8)
Not determined	13 (50)

^a^CT available for current review in 26 patients

^b^These two patients underwent surgery resection of pulmonary sequestration but exact origin of the feeding artery was not identified on CT

Surgical resection was performed in 18 patients (56%). The most common indication for surgery was recurrent respiratory infections in 12 (66%) followed by hemoptysis and pleural effusion in one patient each (Table 4). Four remaining patients underwent surgery for an asymptomatic lung lesion suspected to be PS. Sub-lobar resection was done in 13 (8 ILS, 5 ELS) of 18 (72%) and the remaining five patients underwent lobectomies (all ILS). Postoperative complications were reported in 5 patients (28%; 4 ILS, 1 ELS) and included chylous leak, intraoperative mild bleeding, chronic chest pain, arm numbness and pneumonia. Two of these 5 patients who experienced postoperative complications had been asymptomatic in regard to their lung lesion preoperatively.

**Table 4:** Surgical management data (*n* = 18)

	Number of patients (%)
Indication for surgery	
Recurrent pulmonary infections	12 (66)
Hemoptysis	1 (6)
Pleural effusion	1 (6)
Asymptomatic lung lesions	4 (22)
Type of resection	
Thoracotomy	
Lobectomy	1 (6)
Segmentectomy/sequestrectomy	3 (17)
Wedge resection	2 (11)
VATS	
Lobectomy	4 (22)
Segmentectomy/sequestrectomy	6 (33)
Wedge resection	2 (11)

*VATS* Video-assisted thoracoscopy (VATS)

There was no significant difference in age, gender or sequestration size between surgical and non-surgical patients. However, surgical patients were more often symptomatic at presentation compared to non-surgical (78% vs 29%, *P* = 0.011) (Table 5). There was no surgical mortality (in-hospital).

**Table 5:** Comparison of surgical vs non-surgical patients

Characteristics	Surgical (*n* = 18)	Nonsurgical (*n* = 14)	*P*-value
Age, median (IQR)	41 (27–50)	43 (33–68)	0.218
Sex, n (%)			0.087
Male	7 (39)	10 (71)	
Female	11 (61)	4 (29)	
Sequestration Size, median (IQR)	6.5 (4–7)	6 (3.4–8.9)	0.778
Type of sequestration, n (%)			0.196
Intralobar	13 (72)	13 (93)	
Extralobar	5 (28)	1 (7)	
Presenting symptoms, n (%)			0.011
Asymptomatic	4 (22)	10 (71)	
Symptomatic	14 (78)	4 (29)	

Follow-up data after diagnosis were available in 9 unresected cases and 17 resected cases; the median duration of follow-up was 19 months (IQR 4–26) and 2.5 months (IQR 1–143), respectively, with no complications related to the sequestration reported during follow up.

## Discussion

In this retrospective review of 32 cases of PS diagnosed in adults over a 20-year period in a tertiary care center, we found that 56% of the patients underwent surgical resection. The patients who underwent surgery were more likely to be symptomatic compared to those who did not. Surgical resection of PS was associated with a postoperative complications rate of 28%. The median follow up duration was 19 months for the non-surgical group, and no complications related to the sequestration were reported during the follow up period.

Nearly one-half of the adult patients diagnosed with PS manifested no relevant symptoms. It has been generally believed that most patients should have their PS resected even if they are asymptomatic due to concerns regarding eventual complication, mainly infection of PS. However, this issue remains debatable since data regarding the long-term clinical course and outcome of those with unresected PS are sparse, particularly in the adult population. Our study cohort included adults in their third to seventh decades of life without symptoms referable to the presence of PS and no relevant symptoms or events occurred during follow-up of patients with unresected PS.

Petersen et al. reviewed the literature for patients above the age of 40 with ILS and found 15 cases including two patients from their own medical center [6]. Most of these adult patients underwent surgical resection of their ILS. The largest study in the literature on PS is from China where Wei et al. reported 2625 cases of PS including 132 adult patients. However, their report does not describe how many of their adult PS patients underwent surgical resection, associated surgical outcome, nor clinical course of patients who did not undergo surgical resection [7]. In a study by Makhija et al., 102 older patients (age 4 to 80 years) with congenital cystic lung disease undergoing surgical management were reported and included 20 with PS (20%); postsurgical complication rate of 9.8% for the entire cohort was reported [5].

Berna et al. studied 26 adult patients with ILS all of whom underwent surgical resection [11]. Hemoptysis or recurrent infection was present in 54%. All 26 patients underwent surgical resection of their PS including 20 patients (77%) who underwent lobectomies. Postoperative complication rate was 25% and included pleural empyema, hemoptysis, prolonged air leak, arrhythmia, and fistulae. All patients were alive and well at long-term follow-up (mean 36.5 months).

In our cohort, 56% of patients underwent surgical resection for various indications; the most common indication was recurrent respiratory infection although it was often difficult to prove the relationship between those infections and the sequestration. None of our patients had experienced massive hemoptysis and only three patients (9%) described mild hemoptysis.

The surgical resection of sequestration carries the risk of complications; the surgical complication rate in our cohort was 28% which included chylous leak, intraoperative mild bleeding, chronic chest pain, arm numbness and pneumonia. No surgical mortality occurred. These results are similar to those reported by Berna et al [11].

There are limitations to this study. The retrospective nature of this study limited the extent of data that could be retrieved including preceding symptoms and the exact relationship to the PS. The number of study subjects was modest due to the rarity of PS encountered in the adult population. Nonetheless, our data provide additional insight beyond what is currently available in the literature regarding the clinical course of PS in adults, particularly those who choose not to undergo surgical resection.

## Conclusions

Nearly one-half of adult patients with pulmonary sequestration present with no relevant symptoms. The decision regarding surgical resection needs to weigh various factors including clinical manifestations related to PS, risk of surgical complications, comorbidities, and individual patient preferences.

## Abbreviations

CT: Computed tomography
ELS: Extralobar sequestration
ILS: Intralobar sequestration
IQR: Interquartile range
PS: Pulmonary sequestration
